# WITHDRAWN: Elevated glucose promotes DNA replication and cancer cell growth through pRB-E2F1

**DOI:** 10.21203/rs.3.rs-3126261/v2

**Published:** 2023-12-01

## Abstract

The full text of this preprint has been withdrawn by the authors due to author disagreement with the posting of the preprint. Therefore, the authors do not wish this work to be cited as a reference. Questions should be directed to the corresponding author.

## Full Text Availability

The license terms selected by the author(s) for this preprint version do not permit archiving in PMC. The full text is available from the preprint server.

